# Down-regulation of microRNA-144-3p and its clinical value in non-small cell lung cancer: a comprehensive analysis based on microarray, miRNA-sequencing, and quantitative real-time PCR data

**DOI:** 10.1186/s12931-019-0994-1

**Published:** 2019-03-04

**Authors:** Yu-Ji Chen, Yi-Nan Guo, Ke Shi, Hui-Mei Huang, Shu-Ping Huang, Wen-Qing Xu, Zu-Yun Li, Kang-Lai Wei, Ting-Qing Gan, Gang Chen

**Affiliations:** 1 Department of Medical Oncology, Second Affiliated Hospital of Guangxi Medical University, Daxuedong Road, Nanning, Guangxi Zhuang Autonomous Region 530021 People’s Republic of China; 2 Department of Pathology, Second Affiliated Hospital of Guangxi Medical University, Daxuedong Road, Nanning, Guangxi Zhuang Autonomous Region 530021 People’s Republic of China; 3 Department of Pathology, First Affiliated Hospital of Guangxi Medical University, Shuangrong Road, Nanning, Guangxi Zhuang Autonomous Region 530021 People’s Republic of China

**Keywords:** MiR-144-3p, Non-small cell lung cancer, Microarray, miRNA-sequencing, Quantitative real-time PCR

## Abstract

**Background:**

Previous studies have shown that miR-144-3p might be a potential biomarker in non-small cell lung cancer (NSCLC). Nevertheless, the comprehensive mechanism behind the effects of miR-144-3p on the origin, differentiation, and apoptosis of NSCLC, as well as the relationship between miR-144-3p and clinical parameters, has been rarely reported.

**Methods:**

We investigated the correlations between miR-144-3p expression and clinical characteristics through data collected from Gene Expression Omnibus (GEO) microarrays, the relevant literature, The Cancer Genome Atlas (TCGA), and real-time quantitative real-time PCR (RT-qPCR) analyses to determine the clinical role of miR-144-3p in NSCLC. Furthermore, we investigated the biological function of miR-144-3p by Gene Ontology (GO), Kyoto Encyclopedia of Genes and Genomes (KEGG) analyses. Protein-protein interaction (PPI) network was created to identify the hub genes.

**Results:**

From the comprehensive meta-analysis, the combined SMD of miR-144-3p was − 0.95 with 95% CI of (− 1.37, − 0.52), indicating that less miR-144-3p was expressed in the NSCLC tissue than in the normal tissue. MiR-144-3p expression was significantly correlated with stage, lymph node metastasis and vascular invasion (all *P* <  0.05). As for the bioinformatics analyses, a total of 37 genes were chosen as the potential targets of miR-144-3p in NSCLC. These promising target genes were highly enriched in various key pathways such as the protein digestion and absorption and the thyroid hormone signaling pathways. Additionally, PPI revealed five genes—C12orf5, CEP55, E2F8, STIL, and TOP2A—as hub genes with the threshold value of 6.

**Conclusions:**

The current study validated that miR-144-3p was lowly expressed in NSCLC. More importantly, miR-144-3p might function as a latent tumor biomarker in the prognosis prediction for NSCLC. The results of bioinformatics analyses may present a new method for investigating the pathogenesis of NSCLC.

## Introduction

Lung cancer (LC) is recognized as a life-threatening malady as the incidence and mortality rates are ranked second among all neoplasms worldwide [1]. Accounting for 85% of all diagnosed LC cases, non-small cell lung cancer (NSCLC) is divided mainly into lung squamous cell carcinoma (LUSC), lung adenocarcinoma (LUAD), and large cell carcinoma (LCC) [2]. Currently, the main therapy for NSCLC is a combination of surgery and chemotherapy [3]. Although great progress has been made in the early detection, diagnosis, and targeted treatments of NSCLC, the five-year survival rates are still low, varying from 4 to 17% depending on regional differences and the disease stage [3–5]. Patients with severer tumor and comorbidity burdens are at a higher risk of death from not receiving effective or specific treatments [6]. Thus, the understanding of the molecular mechanisms in NSCLC and the identification of new therapeutic targets are crucial.

MiRNAs are single-stranded ncRNAs of approximately 20 nucleotides in length. They play an important role in the regulation of gene expression by post-transcriptionally binding with the mRNAs of target genes [7]. Participating in cellular differentiation and homeostasis, miRNAs play pivotal roles in cancer [8]. Tissue-specific miRNAs act as novel potential biomarkers in the diagnosis, treatment, and prognosis of cancer [9, 10]. Recently, the effects of miRNAs on oncogenesis and tumor progression have been receiving a great deal of attention.

MicroRNA-144-3p (miR-144-3p), having various functions in different types of cancers, is one of the miRNAs related to cancer. MiR-144-3p acts as a suppressive factor in laryngeal squamous cell carcinoma [11], gastric cancer [12], hepatocellular carcinoma [13], and pancreatic cancer [14]. However, it is an oncogene in renal carcinoma [15], nasopharyngeal carcinoma [16], and colorectal cancer [17]. Previous studies have also implicated that miR-144-3p is involved in cell proliferation, apoptosis, and autophagy by targeting the TP53-inducible glycolysis and apoptosis regulator (TIGAR) in LC [18]. The down-regulation of miR-144-3p results in metabolic alterations of LC cells by regulating the glucose transporter 1(GLUT1) [19]. Previous studies have shown that miR-144-3p might be a biomarker and target with great potential. Nevertheless, the comprehensive mechanism behind the effects of miR-144-3p on the origin, differentiation, and apoptosis of NSCLC, as well as the relationship between miR-144-3p and clinical parameters, has been rarely reported.

This study investigated the correlations between miR-144-3p expression and clinical characteristics through data collected from Gene Expression Omnibus (GEO) microarrays, the relevant literature, The Cancer Genome Atlas (TCGA), and real-time quantitative real-time PCR (RT-qPCR) analyses to determine the clinical role of miR-144-3p in NSCLC. The latent mechanism of action in NSCLC was subsequently examined by using 12 predictive programs to forecast the genes targeted by miR-144-3p. In addition, bioinformatics analyses, which included Gene Ontology (GO), Kyoto Encyclopedia of Genes and Genomes (KEGG), and protein–protein interaction (PPI) network analyses, were performed.

## Materials and methods

### Data collection

**Fig. 1:**
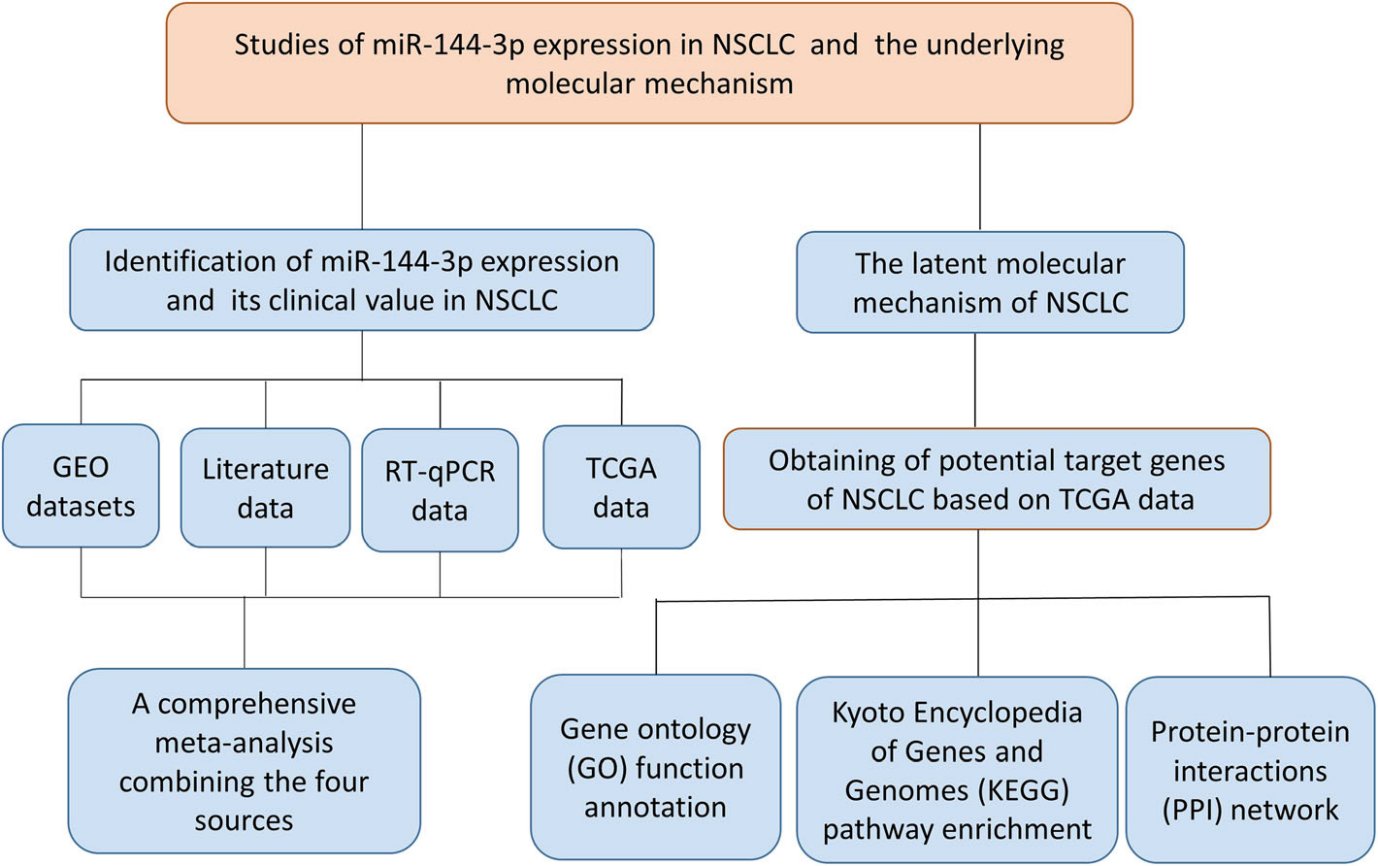
The flow diagram of this study. Note: The workflow indicates that a comprehensive meta-analysis was performed to confirm microRNA-144-3p expression in non-small cell lung cancer and that a bioinformatics analysis was conducted to investigate the latent molecular mechanism

A microarray search of miR-144-3p in NSCLC was conducted in the GEO database (http://www.ncbi.nlm.nih.gov/geo/) with the following keywords: (MicroRNA OR “Micro RNA” OR “non-coding RNA” OR ncRNA OR “small RNA” OR miRNA) AND (Lung OR pulmonary) AND (cancer OR tumor OR neoplasm OR malignancy OR carcinoma OR adenocarcinoma OR AC OR SCC OR NSCLC). The entry type was restricted to “series,” and the organism was filtered by “*Homo sapiens*.” The criteria for inclusion were as follows: (1) patients diagnosed with NSCLC and its subtypes were investigated; (2) cancerous and noncancerous samples were involved; (3) the healthy and malignant groups included at least three samples in the form of tissue, blood, or plasma; (4) and the expression profiling data for miR-144-3p were available. Related studies were retrieved from the PubMed, Google Scholar, China National Knowledge Infrastructure (CNKI), Chongqing VIP electronic (VIP) and Chinese Wanfang databases. Figure 1 shows the workflow for the study.

### MicroRNA-144-3p expression data from the Cancer genome atlas

TCGA (https://cancergenome.nih.gov/) was used for obtaining detailed information about the expression value of miR-144-3p in NSCLC and noncancerous samples. The differences in the miR-144-3p expression in the NSCLC samples and the normal controls were calculated with IBM SPSS Statistics V22.0 software.

### Quantitative real-time PCR

For the current study, 125 matched samples were provided by the Department of Pathology of the First Affiliated Hospital of Guangxi Medical University. Formalin fixation and paraffin embedding (FFPE) were performed to preserve the specimens. This aspect of the research was authorized by the hospital’s ethics committee. Next, the miR-144-3p expression in 125 paired clinical samples was detected by RT-qPCR with the Applied Biosystems 7900HT Fast Real-Time PCR System software. The miR-144-3p sequence was as follows: UACAGUAUAGAUGAUGUACU. The formula for 2-Δcq was used for the calculation of the miR-144-3p expression value.

### Statistical analysis and comprehensive meta-analysis

After miR-144-3p was log_2_-transformed, the expression profiling information was used to calculate the number, mean (M) and standard deviation (SD) for each control and experimental group with IBM SPSS Statistics V22.0 software. In addition, Stata 12.0 software was used for performing a comprehensive meta-analysis of data aggregated from multiple sources (microarray, literature, miRNA sequencing, and RT-qPCR). The analysis of the miR-144-3p expression in the NSCLC and tumor-free specimens was displayed on forest plots that illustrate the standardized mean difference (SMD) and the 95% confidential interval (CI). The chi-squared test of Q and the I^2^ statistic were calculated to assess heterogeneity across the studies and to determine the appropriateness of applying either a random effects model or fixed effects model to the pooling process. To measure publication bias, Egger’s and Begg’s tests and a funnel plot, for which significance was *p* <  0.05, were performed.

### Latent targets of microRNA-144-3p in non-small cell lung cancer

MiRWALK2.0, an online archive of data on miRNA-target interactions [20], was mined to forecast the genes targeted by miR-144-3p. In total, 12 servers with miRWalk, miRMap, MicroT4, miRNAMap, TargetScan PICTAR2, miRBridge, PITA, miRanda, RNAhybrid, miRDB, RNA22 were used. Only those genes projected by more than six of the servers were recognized as target genes. The high-expressed genes in LUAD and LUSC were acquired through Gene Expression Profiling Interactive Analysis (GEPIA). The overlapping genes among the up-regulated genes in LUAD and LUSC and the predicted target genes, were viewed as promising targets of miR-144-3p in NSCLC. A review of the literature on the specific target genes of miR-144-3p in NSCLC was conducted. The target genes determined by previous studies and the predicted target genes, namely promising target genes, were used in the functional analysis.

### Functional analysis for promising target genes

The GO vocabularies, which include biological processes (BPs), cellular components (CCs), and molecular functions (MFs), were enriched by Metascape (http://metascape.org/gp/). The functional annotation of the underlying target genes was then elucidated by KEGG pathway analysis with Metascape tool. In addition, a PPI network was constructed to reveal the hub genes of the potential target genes on STRING, a web portal for undermining the integrated function of multiple genes [21].

### Expression of hub genes from the Cancer genome atlas and the genotype-tissue expression database

To further confirm the function of hub genes in NSCLC and their relationship to miR-144-3p, a search of TCGA and the Genotype-Tissue Expression (GTEx) database was performed to determine the expression pattern of the hub genes in NSCLC. Box plots of the hub genes in the NSCLC and non-cancer samples were developed through GEPIA.

## Results

### Confirmation of the expression and clinical value of microRNA-144-3p in non-small cell lung cancer, based on gene expression omnibus datasets

#### MicroRNA-144-3p expression in non-small cell lung cancer obtained through gene expression omnibus microarrays

**Fig. 2:**
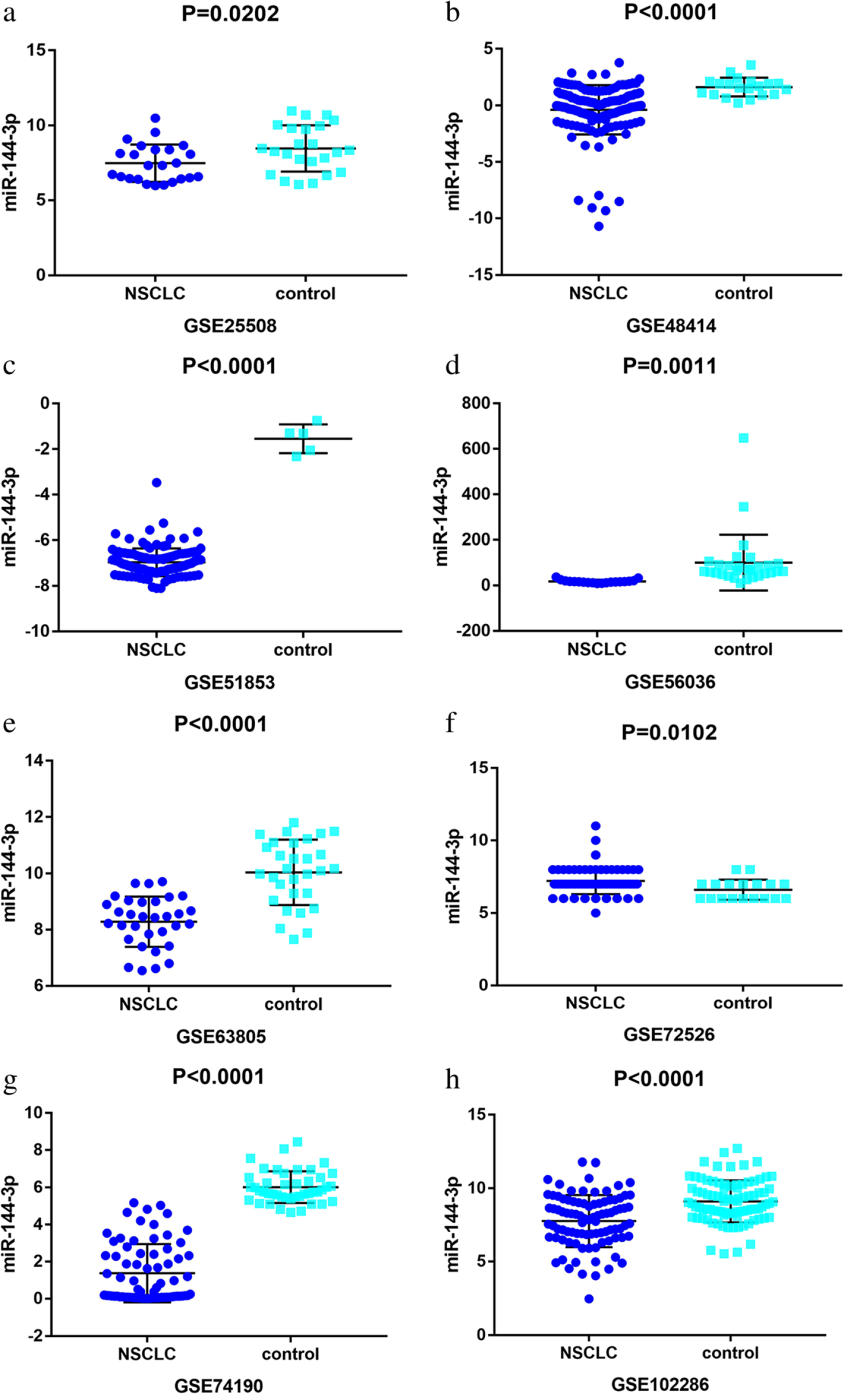
Down-regulation of microRNA-144-3p in in the other microarrays tissues, based on Gene Expression Omnibus datasets. Notes: **a** GSE25508. **b** GSE48414. **c** GSE51853. **d** GSE56036. **e** GSE63805. **f** GSE72526. **g** GSE74190. **h** GSE102286

**Fig. 3:**
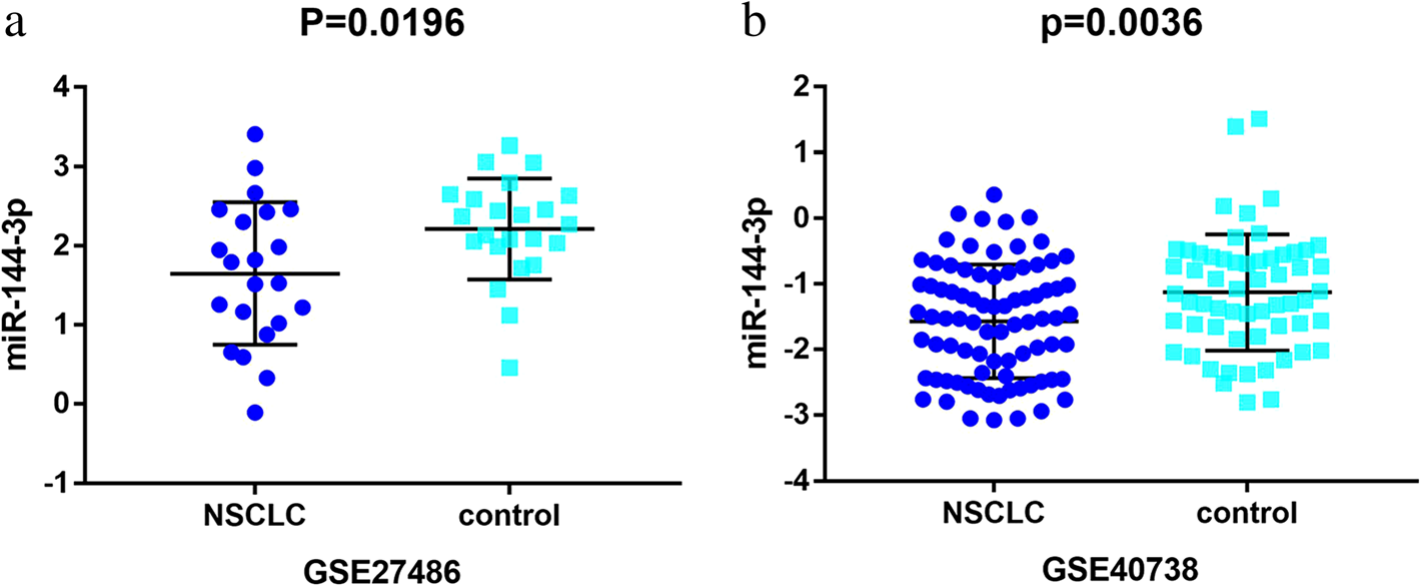
Low expression of miR-144-3p in non-small cell lung cancer chips derived from blood sample . Notes: **a** GSE27486. **b** GSE40738

A total of 19 microarrays from the GEO database met the entry criteria. The features of the included GEO datasets are depicted in Table 1. Of the microarrays, 14 were obtained from tissue, and 5 were derived from blood (GSE27486, GSE40738, GSE64951, GSE93300, and GSE114711). In addition, the expression data from the NSCLC and control groups were collected on the basis of the GEO database. With respect to the data from the tissue samples, the NSCLC groups had a significantly lower level of miR-144-3p expression than the control groups in GSE25508, GSE48414, GSE51853, GSE56036, GSE63805, GSE72526, GSE74190, and GSE102286 (*p* = 0.0202, *p* <  0.0001, *p* <  0.0001, *p* = 0.0011, *p* <  0.0001, *p* = 0.0102, *p* <  0.0001, and *p* <  0.0001, respectively (Fig. 2)). In contrast, no notable distinction in miR-144-3p expression was detected between the NSCLC and the control groups in the other microarrays (GSE14936, GSE29248, GSE36681, GSE47525, GSE53882, and GSE77380). Regarding the data from the blood samples, miR-144-3p expression in NSCLC was found to decrease significantly in GSE27486 and GSE40738 (*p* = 0.0196, *p* = 0.0036, respectively (Fig. 3)).

**Table 1:** Features of the enrolled Gene Expression Omnibus datasets

Accession	GPL	Year	NSCLC			Control			Source
			n	M	SD	n	M	SD	
GSE14936	GPL8879	2012	22	7.8687	0.78175	19	8.0904	0.82617	tissue
GSE25508	GPL7731	2014	24	7.4846	1.24999	24	8.463	1.55049	tissue
GSE27486	GPL11432	2012	22	1.6495	0.90141	23	2.2125	0.64072	blood
GSE29248	GPL8179	2014	6	672.5763	701.2225	6	648.1356	587.8154	tissue
GSE36681	GPL8179	2014	103	9.2339	0.66957	103	9.2783	0.62179	tissue
GSE40738	GPL16016	2017	82	−1.5715	0.86663	59	−1.1291	0.88742	blood
GSE47525	GPL17222	2015	14	2.4786	0.86574	14	2.5643	1.02852	tissue
GSE48414	GPL16770	2015	154	−0.3765	2.18784	20	1.6223	0.82035	tissue
GSE51853	GPL7341	2016	126	−6.9614	0.60881	5	−1.5464	0.63552	tissue
GSE53882	GPL18130	2017	397	1.6467	1.57039	151	1.8168	1.89299	tissue
GSE56036	GPL15446	2017	19	17.7554	7.25245	29	100.0724	121.8101	tissue
GSE63805	GPL18410	2016	32	8.286	0.89384	30	10.0377	1.15923	tissue
GSE64591	GPL18942	2018	100	4.9474	0.08751	100	4.9618	0.09277	blood
GSE72526	GPL20275	2015	67	7.2239	0.91818	18	6.6111	0.6978	tissue
GSE74190	GPL19622	2015	72	1.3728	1.57232	44	6.016	0.85477	tissue
GSE77380	GPL16770	2016	3	1.8937	4.51735	12	4.2684	3.91992	tissue
GSE93300	GPL21576	2017	9	−6.0441	0.99527	4	−6.572	3.92546	blood
GSE102286	GPL23871	2018	91	7.763	1.77768	88	9.1063	1.43636	tissue
GSE114711	GPL18573	2018	19	6.9034	1.33845	7	6.9887	1.37975	blood

*NSCLC* Non-small cell lung cancer, *M* Mean, *SD* Standard deviation

#### Results of meta-analysis of gene expression omnibus datasets

**Fig. 4:**
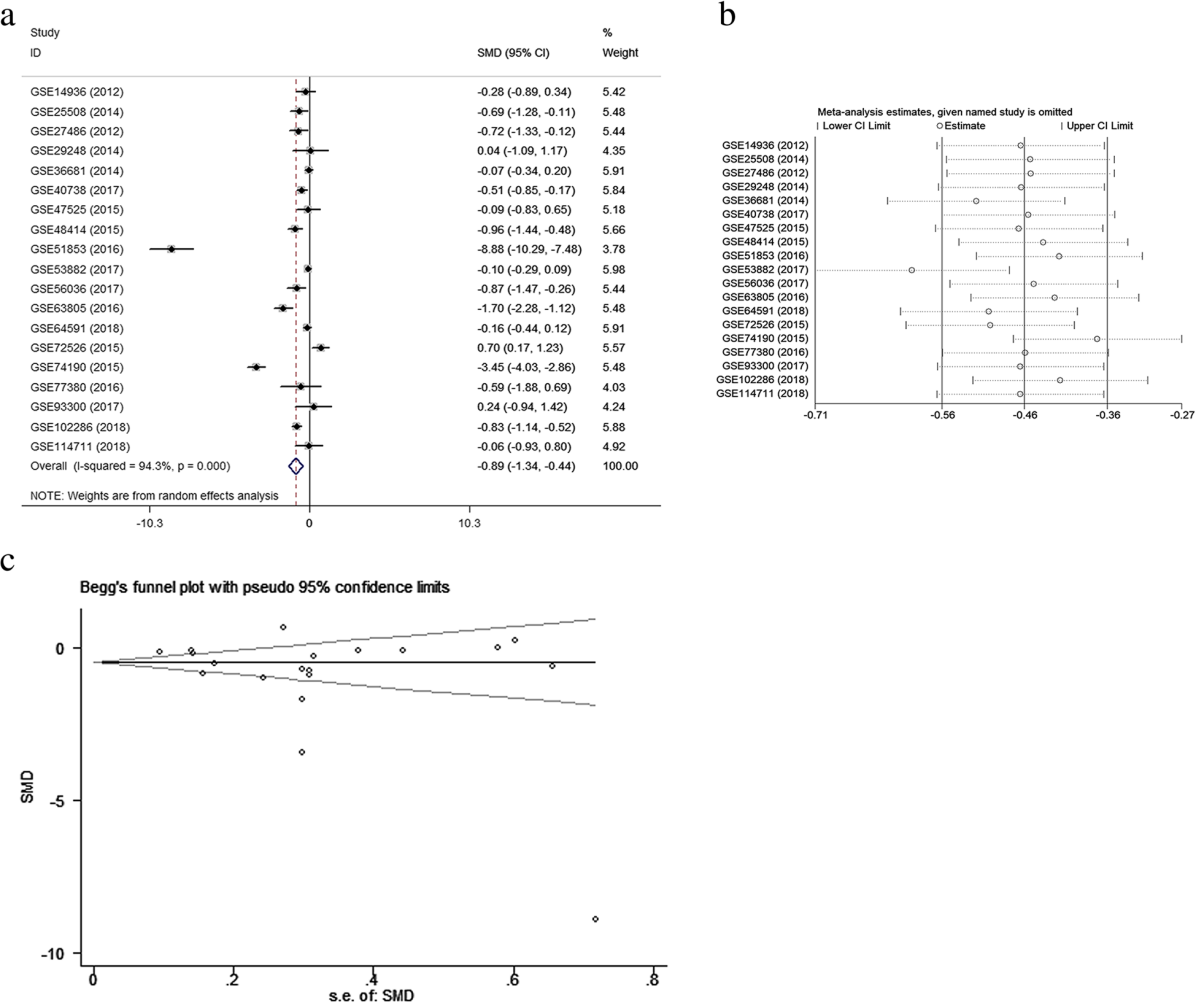
Meta-analysis of Gene Expression Omnibus (GEO) data. Notes: **a** Forest plot of GEO chips. The pooled standard mean deviation of −0.89 (95%: −1.34, − 0.44) with great heterogeneity (I^2^ = 94.3%, *p* = 0.000) showed that microRNA-144-3p expression had markedly reduced in the non-small cell lung cancer tissues. **b** Sensitivity analysis of GEO chips. **c** A funnel plot was applied to evaluate the publication bias of GEO chips (Begg’s test, *p* = 0.363)

A meta-analysis was conducted on the basis of the 19 included microarrays from the GEO database. The results are demonstrated in Fig. 4a. Given the apparent heterogeneity (*p* <  0.05, I^2^ = 94.3%), a random effects model was applied, and remarkable down-regulation (SMD = − 0.89; 95% CI − 1.34, − 0.44; *p* = 0.000) of miR-144-3p was found in the NSCLC groups. A sensitivity analysis was later conducted to explore whether a particular microarray played a vital role in significant heterogeneity (Fig. 4b). After an individual study was removed each time of meta-analysis, the combined effect was compared to the previous one. No study was found to have played a crucial role in any of the enrolled studies.

**Fig. 5:**
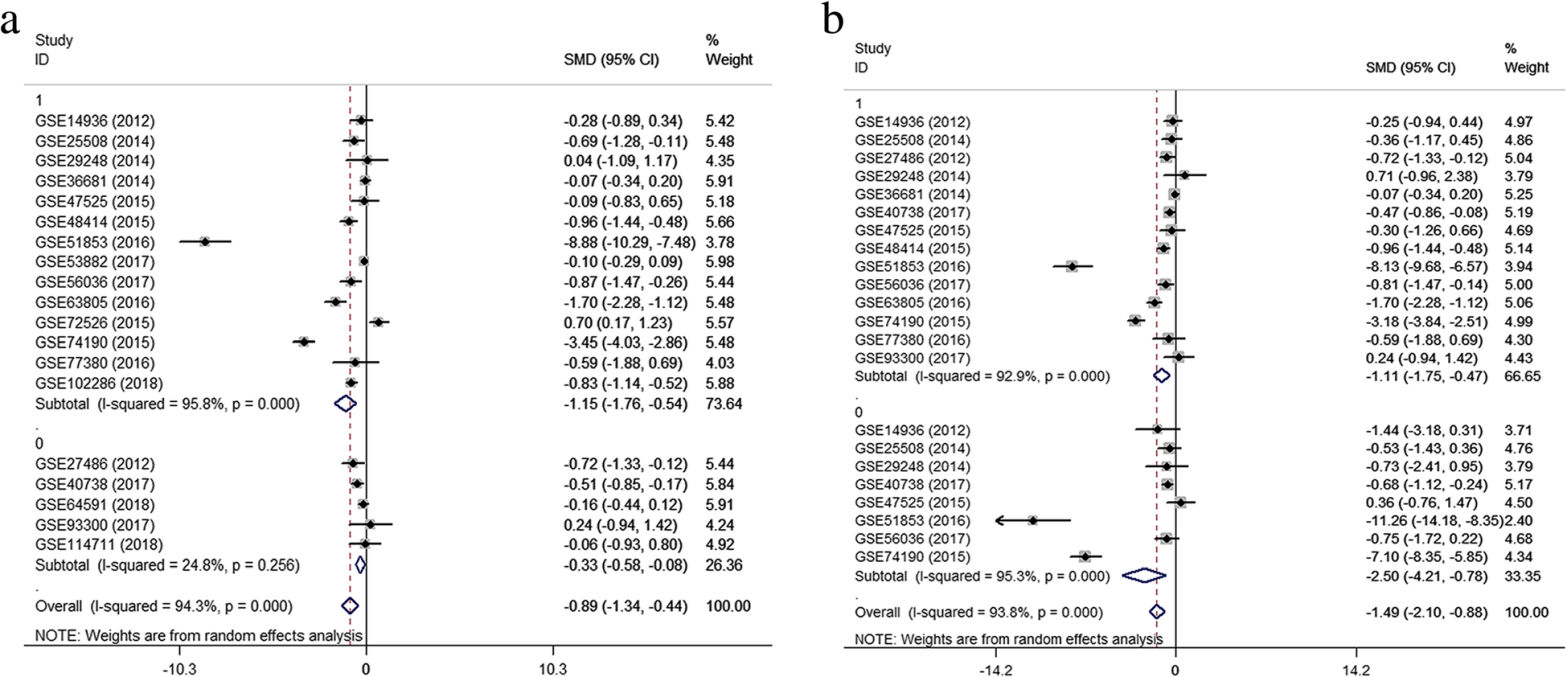
Results of subgroup analyses. Notes: **a** Subgroup analysis based on sample source. The tissue subgroup had significant heterogeneity (I^2^ = 95.8%, *p* = 0.000). (1: tissue, 0: blood) **b** Subgroup analysis based on cancer type. The adenocarcinoma and the squamous cell carcinoma both had significant heterogeneity (1: adenocarcinoma, 0: squamous cell carcinoma)

A funnel plot was generated to estimate publication bias (Fig. 4c). To further clarify the heterogeneity source, a subgroup analysis was performed. It was based on multiple characteristics: sample source (tissue vs. blood), and cancer type (adenocarcinoma vs. squamous cell carcinoma). As is illustrated in Fig. 5, significant heterogeneity was observed in the tissue subgroup (I^2^ = 95.8%, *p* = 0.000). Significant heterogeneity was also found in adenocarcinoma (I^2^ = 92.9%, *p* = 0.000) and squamous cell carcinoma (I^2^ = 95.3%, *p* = 0.000). These results suggest that sample source and cancer type might be sources of heterogeneity.

#### Literature

A review of the literature relevant to miR-144-3p was conducted through searches in the PubMed, Google Scholar, CNKI, VIP, and Wanfang databases. Neither the M nor the SD of miR-144-3p in the NSCLC and normal groups was provided in the literature; therefore, no available data could be obtained from the existing studies.

### Confirmation of the expression and clinical effects of microRNA-144-3p in non-small cell lung cancer, based on the Cancer genome atlas data

#### MicroRNA-144-3p expression and prognostic value in non-small cell lung cancer tissues

**Fig. 6:**
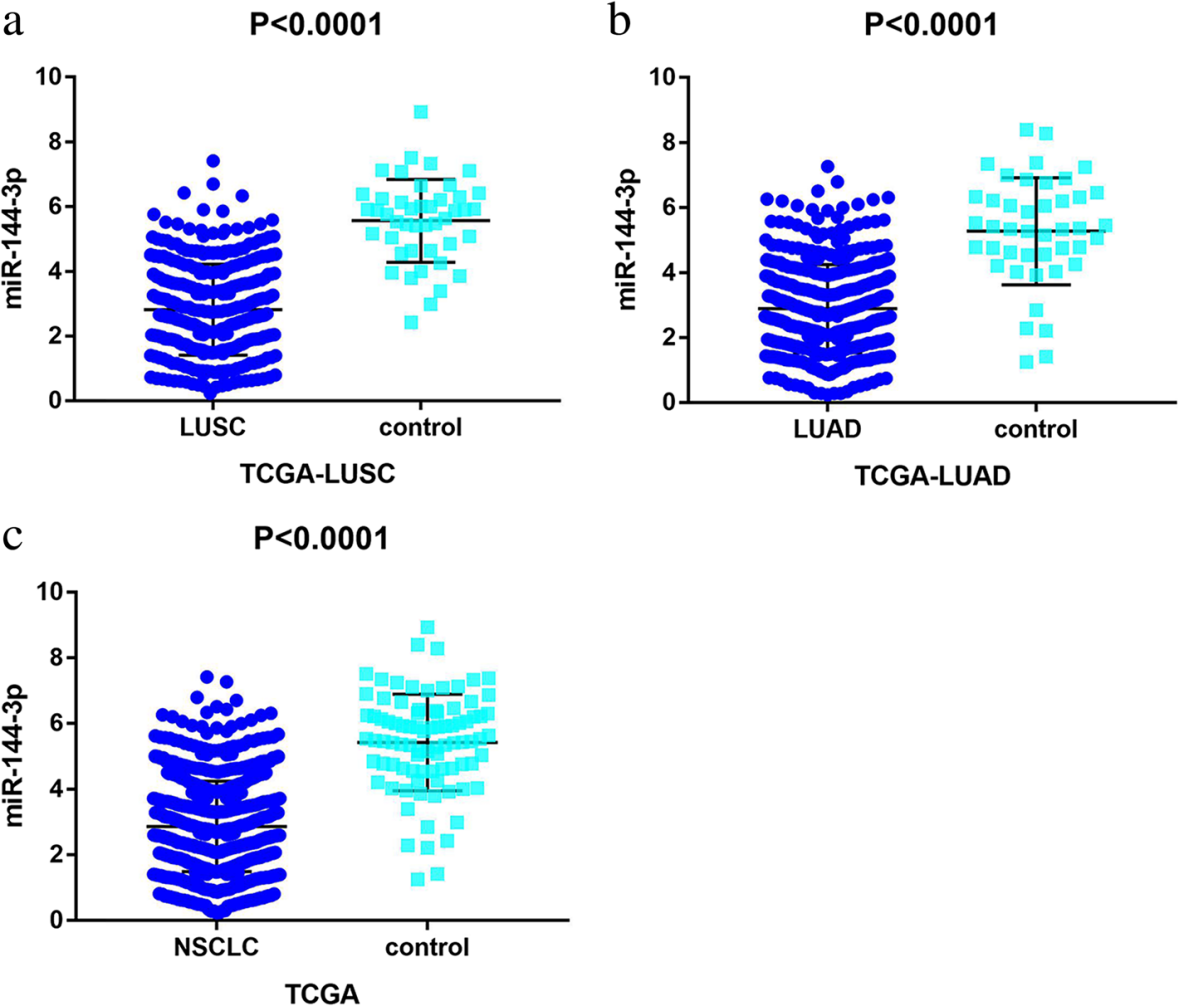
Patterns of microRNA-144-3p expression in non-small cell lung cancer tissues and normal tissues, in accordance with The Cancer Genome Atlas data. Note: **a** MicroRNA-144-3p expression in lung squamous cell carcinoma, **b** lung adenocarcinoma, and **c** non-small cell lung cancer tissues was less than that in normal tissues

**Fig. 7:**
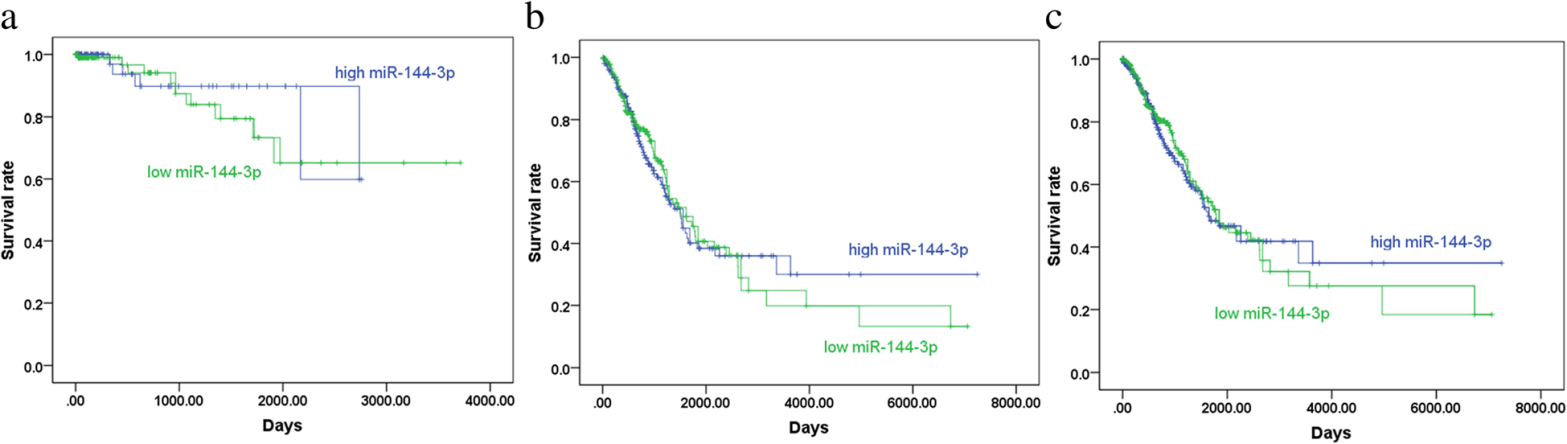
Kaplan–Meier curves for microRNA-144-3p in **a** lung squamous cell carcinoma (LUSC), **b** lung adenocarcinoma (LUAD), and **c** non-small cell lung cancer (NSCLC) tissues, based on The Cancer Genome Atlas data. Notes: The *p* values for the survival curves of LUSC, LUAD, and NSCLC were 0.509, 0.863, and 0.808, respectively. No distinct prognostic differences were observed among them. (green curve: low expression, blue curve: high expression)

TCGA contained 376 samples for LUSC patients and 488 samples for LUAD patients. Regarding LUSC, miR-144-3p expression was remarkably downregulated in comparison with the normal controls (2.8193 ± 1.40600 vs. 5.5678 ± 1.27693, *p* <  0.001 (Fig. 6a and Table 2)). In terms of LUAD, the expression level of miR-144-3p was obviously less than that in the healthy tissue (2.8959 ± 1.35967 vs. 5.2775 ± 1.64708, *p* <  0.001 (Fig. 6b and Table 3)). The data from LUSC and LUAD were combined for further examination of the miR-144-3p expression in NSCLC. As is illustrated in Fig. 6c and Table 4, miR-144-3p was significantly reduced in the NSCLC tissue compared to the non-cancerous lung tissue (2.8632 ± 1.37928 vs. 5.4243 ± 1.4702, *p* <  0.0001). A Kaplan–Meier curve was later used to identify the effects of the expression of miR-144-3p on survival time. As is shown in Fig. 7, the *p* values for the three Kaplan–Meier curves were all greater than 0.05, thus indicating no significant difference in survival time between the group with low levels of miR-144-3p and the one with high levels.

**Table 2:** Association between microRNA-144-3p expression and clinicopathological parameters in lung squamous cell carcinoma, based on The Cancer Genome Atlas data

Clinicopathological feature		n	mean ± SD	*p*-value
Tissue	Adjacent non-cancerous tissue	44	5.5678 ± 1.27693	< 0.001^*^
	LUSC	332	2.8193 ± 1.40600	
Age (years)	< 60	59	2.7458 ± 1.32704	0.739
	≥60	267	2.8106 ± 1.42109	
Gender	Female	84	2.8195 ± 1.49697	0.999
	Male	248	2.8192 ± 1.37698	
Tumor location	Central lung	106	2.7551 ± 1.27303	0.848
	Peripheral lung	73	2.7968 ± 1.51724	
Stage	Stage I-II	276	2.8878 ± 1.39556	0.04^*^
	Stage III-IV	53	2.4469 ± 1.41079	
T	T1-T2	262	2.9023 ± 1.40854	0.035^*^
	T3-T4	70	2.5088 ± 1.36178	
N	No	213	2.8637 ± 1.43682	0.302
	Yes	113	2.6987 ± 1.33482	
M	No	252	2.7345 ± 1.38222	0.895
	Yes	3	2.9144 ± 2.09106	

*SD* Standard deviation, *LUSC* Lung squamous cell carcinoma

^*^p < 0.05 was considered statistically significant

**Table 3:** Association between microRNA-144-3p expression and clinicopathological parameters in lung adenocarcinoma, based on The Cancer Genome Atlas data

Clinicopathological		n	mean ± SD	*p* value
Tissue	Adjacent non-cancerous tissue	43	5.2775 ± 1.64708	< 0.001^*^
	LUAD	445	2.8959 ± 1.35967	
Age (years)	< 60	120	3.0042 ± 1.40207	0.168
	≥60	306	2.7979 ± 1.33460	
Gender	Female	239	2.9011 ± 1.33672	0.931
	Male	206	2.8898 ± 1.38905	
Tumor location	Central lung	54	2.5665 ± 1.1857	0.382
	Peripheral lung	113	2.747 ± 1.3564	
Stage	Stage I-II	349	2.8191 ± 1.33042	0.031^*^
	Stage III-IV	91	3.1868 ± 1.45395	
T	T1-T2	385	2.9199 ± 1.35515	0.133
	T3-T4	57	2.6359 ± 1.3106	
N	No	291	2.8546 ± 1.35977	0.553
	Yes	145	2.9367 ± 1.35784	
M	No	283	2.8058 ± 1.31875	0.126
	Yes	19	3.3343 ± 1.4003	

*SD* Standard deviation, *LUAD* Lung adenocarcinoma

^*^p < 0.05 was considered statistically significant

**Table 4:** Association between microRNA-144-3p expression and clinicopathological parameters in non-small cell lung cancer, based on The Cancer Genome Atlas data

Clinicopathological feature		n	mean ± SD	*p*-value
Tissue	Adjacent non-cancerous tissue	87	5.4243 ± 1.4702	< 0.001^*^
	LUSC+LUAD	777	2.8632 ± 1.37928	
Age (years)	< 60	179	2.919 ± 1.37944	0.330
	≥60	573	2.8038 ± 1.37438	
Gender	Female	323	2.8799 ± 1.37826	0.776
	Male	454	2.8513 ± 1.38139	
Tumor location	Central lung	160	2.6915 ± 1.24372	0.604
	Peripheral lung	186	2.7666 ± 1.41787	
Stage	Stage I-II	625	2.8494 ± 1.3589	0.630
	Stage III-IV	144	2.9145 ± 1.47731	
T	T1-T2	647	2.9128 ± 1.37596	0.008^*^
	T3-T4	127	2.5658 ± 1.33528	
N	No	504	2.8585 ± 1.39145	0.803
	Yes	258	2.8324 ± 1.35039	
M	No	535	2.7722 ± 1.34822	0.124
	Yes	22	3.277 ± 1.45564	

*SD* Standard deviation, *LUAD* Lung adenocarcinoma, *LUSC* Lung squamous cell carcinoma

^*^p < 0.05 was considered statistically significant

#### Relationships between microRNA-144-3p and clinical pathology of non-small cell lung cancer, based on the Cancer genome atlas data

As can be seen in Tables 2 and 3, the clinical characteristics of 332 LUSC patients and 445 LUAD patients were downloaded from TCGA. Regarding LUSC, a significant difference in miR-144-3p was found for stage (*p* = 0.040) and primary tumor (T) (*p* = 0.035). LUSC patients in Stages III–IV (2.4469 ± 1.41079) had a lower expression of miR-144-3p than those in Stages I–II (2.8878 ± 1.39556). The miR-144-3p expression of LUSC in T3–T4 (2.5088 ± 1.36178) was more significantly decreased than T1–T2 (2.9023 ± 1.40854). In terms of LUAD, a significant difference of miR-144-3p expression was observed for stage (*p* = 0.031). Patients in Stages III–IV (3.1868 ± 1.45395) had higher expression values of miR-144-3p. The data on LUAD and LUSC, based on TCGA, were pooled for further validation. As is illustrated in Table 4, the significance in the statistics for the T stage was based on the lower miR-144-3p expression in patients in T3–T4 (*p* <  0.05).

### Quantitative real-time PCR analysis

#### The microRNA-144-3p expression and its significance in non-small cell lung cancer prognoses

**Fig. 8:**
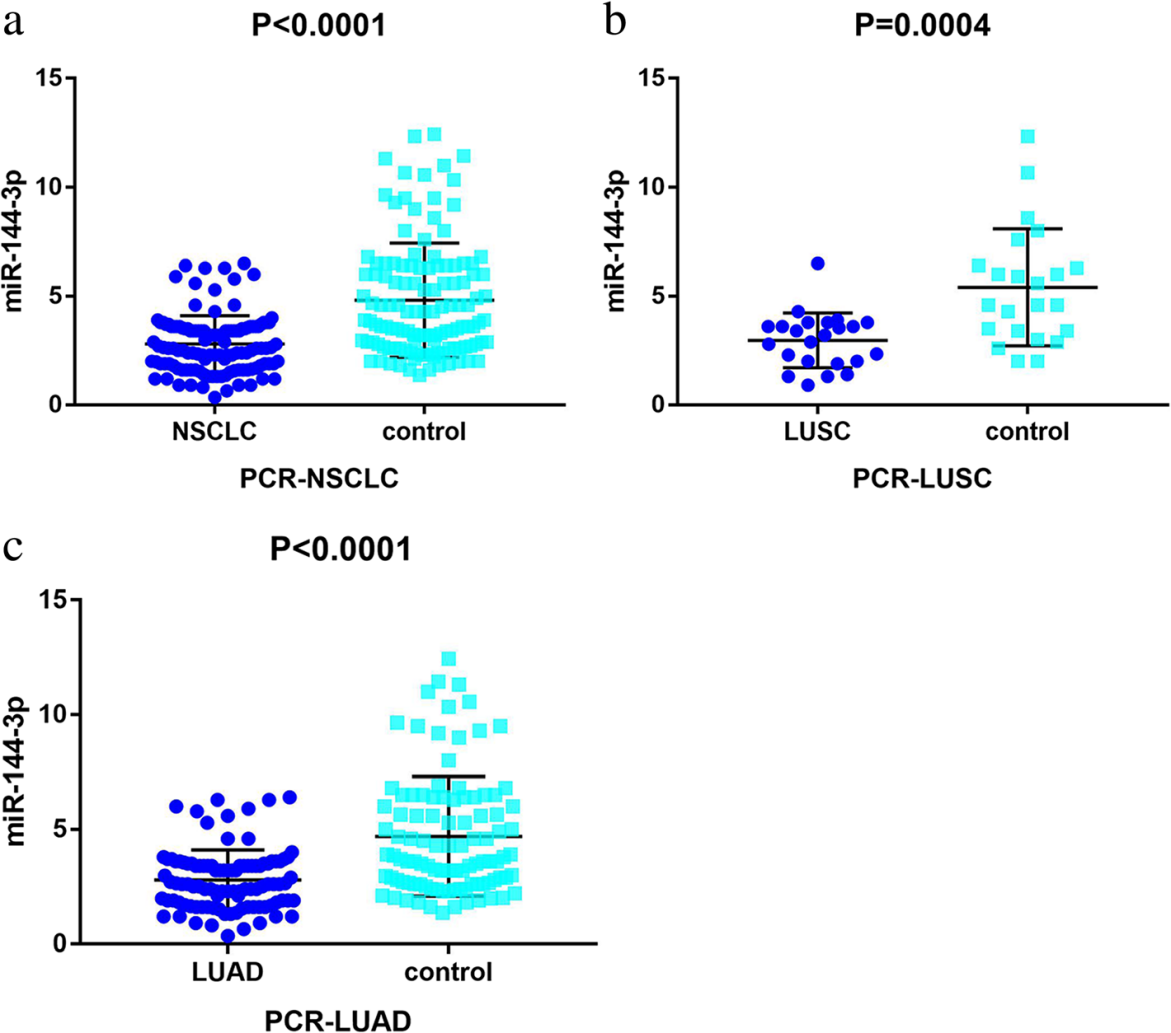
Patterns of microRNA-144-3p expression in clinical samples, based on quantitative real-time PCR data. Note: Lowly expressed microRNA-144-3p was observed in **a** 23 lung squamous cell carcinoma tissues, **b** 101 lung adenocarcinoma tissues, and **c** 125 non-small cell lung cancer tissues

**Fig. 9:**
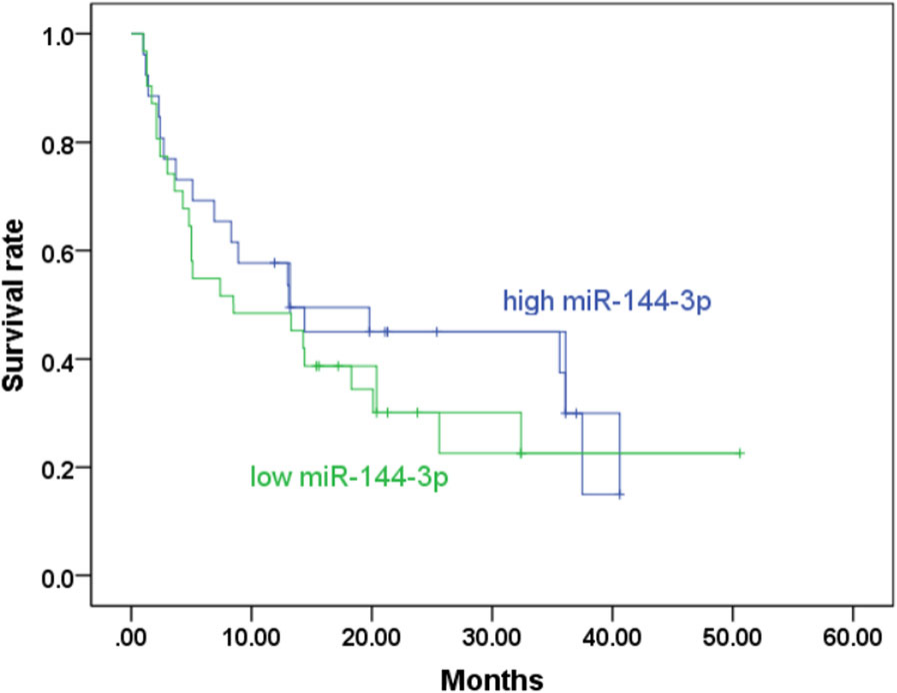
A Kaplan–Meier curve for microRNA-144-3p in clinical lung adenocarcinoma samples. Note: The *p* value of the Kaplan–Meier curve for the clinical lung adenocarcinoma patients was 0.397, highlighting a trend of possibly longer overall survival rates in the high microRNA-144-3p group. (green curve: low expression, blue curve: high expression)

Using RT-qPCR, the clinical expression value of miR-144-3p in 125 matched tissues was evaluated. As is illustrated in Fig. 8a and Table 5, the NSCLC samples exhibited a significantly lower expression level of miR-144-3p than the non-cancerous samples (2.808 ± 1.303 vs. 4.813 ± 2.618, *p* <  0.001). Next, miR-144-3p expression was then analyzed for LUAD and for LUSC. As is shown in Fig. 8b and c, unlike what was found in the adjacent non-cancerous tissue, apparently lowly expressed miR-144-3p was observed in both LUSC and LUAD (*p* = 0.0004, *p* <  0.0001). A Kaplan–Meier curve was generated to assess whether miR-144-3p is appropriate for the prognosis prediction of LUAD. As is depicted in Fig. 9, the LUAD patients who exhibited lower expression values of miR-144-3p might have worse outcomes (*p* = 0.397).

**Table 5:** Associations between microRNA-144-3p expression and clinicopathological features in non-small cell lung cancer based on quantitative real-time PCR data

Clinicopathological feature		n	mean ± SD	*p* value
Tissue	Adjacent non-cancerous tissue	125	4.813 ± 2.618	< 0.001^*^
	NSCLC	125	2.808 ± 1.303	
Age (years)	< 60	57	2.7879 ± 1.33904	0.874
	≥60	68	2.8254 ± 1.28196	
Gender	Female	50	2.9786 ± 1.40038	0.247
	Male	75	2.6948 ± 1.23055	
Tumor size (cm)	≤3	60	2.9242 ± 1.44526	0.346
	> 3	65	2.7014 ± 1.15768	
Smoke	No	38	3.2489 ± 1.48252	0.166
	Yes	30	2.7767 ± 1.29623	
Lymph node metastasis	No	56	3.0875 ± 1.38065	0.033^*^
	Yes	69	2.5817 ± 1.19935	
Vascular invasion	No	90	3.0682 ± 1.24395	< 0.001^*^
	Yes	35	2.1400 ± 1.22630	
TNM	I-II	54	2.9615 ± 1.27286	0.251
	III-IV	71	2.6918 ± 1.32268	
Histological type	ADC	101	2.7917 ± 1.30837	0.561
	SCC	23	2.9643 ± 1.26398	
	LCLC	1	0.9000	

*SD* Standard deviation, *NSCLC* Non-small cell lung cancer, *ADC* Adenocarcinoma, *SCC* Squamous cell carcinoma, *LCLC* Large-cell lung carcinoma

^*^p < 0.05 was considered statistically significant

#### Correlations between microRNA-144-3p expression and clinical characteristic for non-small cell lung cancer patients

The miR-144-3p expression in NSCLC cases was significantly different in lymph node metastasis and vascular invasion (Table 5). The lower value of miR-144-3p was found in patients with lymph node metastasis but not in those without it (Table 5). Patients with vascular invasion maintained a miR-144-3p value of 2.1400 ± 1.2263, and the expression level of those without vascular invasion was 3.0682 ± 1.24395 (Table 5). To further validate the correlation of miR-144-3p and clinicopathological characteristics, the NSCLC cases were divided into LUSC and LUAD groups. For the LUSC group (Table 6), no statistical differences were seen for smoking, vascular invasion, or lymph node metastasis. However, for LUAD, the statistical analyses indicated significant differences for smoking, vascular invasion, and lymph node metastasis. In comparison to patients without vascular invasion, those with vascular invasion had lower miR-144-3p expression values. The miR-144-3p expression in patients with a smoking habit was significantly down-regulated over that of patients without the habit (*p* = 0.027, Table 7). Besides, the miR-144-3p expression in NSCLC patients who were considered to have lymph node metastasis was markedly reduced.

**Table 6:** Associations between microRNA-144-3p expression and clinicopathological features in lung squamous cell carcinoma, based on quantitative real-time PCR data

Clinicopathological feature		n	mean ± SD	*p* value
Tissue	Adjacent non-cancerous tissue	23	5.405 ± 2.684	0.0004^*^
	LUSC	23	2.964 ± 1.264	
Age (years)	< 60	15	2.872 ± 1.39748	0.611
	≥ 60	8	3.1375 ± 1.03086	
Gender	Female	5	2.976 ± 0.6827	0.974
	Male	18	2.9611 ± 1.39922	
Tumor size (cm)	≤ 3	7	3.02 ± 1.79763	0.915
	> 3	16	2.94 ± 1.02398	
Smoke	No	12	2.6733 ± 1.1468	0.263
	Yes	11	3.2818 ± 1.36222	
Lymph node metastasis	No	11	3.0436 ± 1.48816	0.784
	Yes	12	2.8917 ± 1.08163	
Vascular invasion	No	20	2.919 ± 0.99752	0.667
	Yes	3	3.2667 ± 2.82194	
TNM	I–II	10	2.978 ± 0.89643	0.963
	III–IV	13	2.9538 ± 1.52513	

*SD* Standard deviation, *LUSC* Lung squamous cell carcinoma

^*^p < 0.05 was considered statistically significant

**Table 7:** Associations between microRNA-144-3p expression and clinicopathological features in lung adenocarcinoma, based on quantitative real-time PCR data

Clinicopathological feature		n	mean ± SD	*p*-value
Tissue	Adjacent non-cancerous tissue	101	4.693 ± 2.607	< 0.001^*^
	LUAD	101	2.792 ± 1.308	
Age (years)	< 60	41	2.8032 ± 1.31708	0.942
	≥60	60	2.7838 ± 1.31347	
Gender	Female	45	2.9789 ± 1.46340	0.211
	Male	56	2.6413 ± 1.16082	
Tumor size (cm)	≤3	53	2.9115 ± 1.41270	0.332
	> 3	48	2.6594 ± 1.18327	
Smoke	No	26	3.5146 ± 1.56261	0.027^*^
	Yes	18	2.5722 ± 1.16539	
Lymph node metastasis	No	45	3.0982 ± 1.3707	0.037^*^
	Yes	56	2.5454 ± 1.21274	
Vascular invasion	No	70	3.1109 ± 1.30906	< 0.001^*^
	Yes	31	2.0710 ± 0.99515	
TNM	I-II	44	2.9577 ± 1.35229	0.269
	III-IV	57	2.6635 ± 1.27056	

*SD* Standard deviation, *LUAD* Lung adenocarcinoma

^*^p < 0.05 was considered statistically significant

#### Meta-analysis of combination of gene expression omnibus, the Cancer genome atlas, and quantitative real-time PCR data

**Fig. 10:**
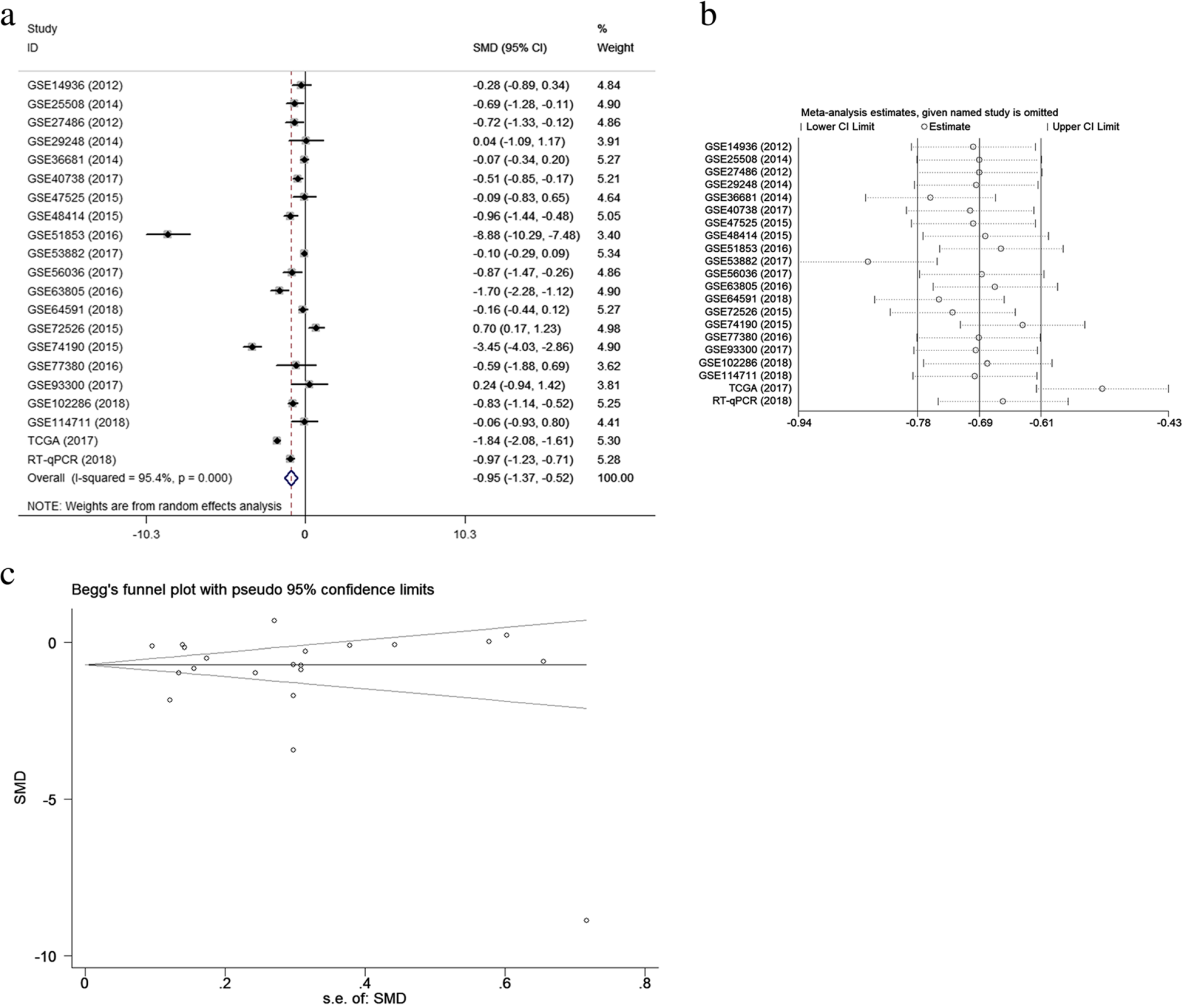
A comprehensive meta-analysis based on Gene Expression Omnibus (GEO), The Cancer Genome Atlas (TCGA), and quantitative real-time PCR (RT-qPCR) datasets. **a** Forest plot of microRNA-144-3p expression data from GEO, TCGA, and RT-qPCR datasets. With the random effects model, the I^2^ value was 95.4%. **b** Sensitivity analysis of GEO, TCGA, and RT-qPCR datasets. **c** The evaluation of the publication bias of the GEO, TCGA, and RT-qPCR datasets (Begg’s test, *p* = 0.833)

Because no relevant studies were found, data from three sources (microarrays, miRNA-sequencing, and RT-qPCR) containing 2264 NSCLC samples and 968 non-cancerous samples were extracted for integrated meta-analyses with a random effects model because of high heterogeneity (I^2^ = 95.4%, *p* = 0.000). The differences in individuals, NSCLC subtype, and sample source were considered the sources of heterogeneity. As is shown in Fig. 10a, the combined SMD of miR-144-3p was − 0.95 with 95% CI of (− 1.37, − 0.52), indicating that less miR-144-3p was expressed in the NSCLC tissue than in the normal tissue. The sensitivity analysis (Fig. 10b) indicated significant differences among the studies; however, no specific study had a significant effect on high heterogeneity. The evaluation of publication bias was performed with Begg’s and Egger’s tests and a funnel plot (Fig. 10c). In general, the funnel plot was symmetrical, and the *p* values obtained from the Begg’s and Egger’s tests were 0.833 and 0.335, respectively. In sum, the results indicate that publication bias for the studies was controlled passably.

## Bioinformatic analyses

### Promising target genes collection

From miRWALK2.0, 1635 genes targeted by miR-144-3p in NSCLC predicted by more than six algorithms were obtained. A total of 1109 overexpressed genes in LUAD were collected on the basis of GEPIA. In addition, 1922 overexpressed genes in LUSC were acquired from GEPIA. After intersection, 34 predicted target genes were selected. Four specific targets of miR-144-3p were verified in previous studies related to LC (Table 8). The TIGAR is also known as the C12orf5 gene. Accordingly, a total of 37 potential target genes were collected.

**Table 8:** Identified target genes derived from the literature

validated target gene	PMID
BLACAT1	PMID: 28885863
GLUT1	PMID: 27313692
TIGAR	PMID: 25660220
ZEB1	PMID: 26191328

### Gene ontology and Kyoto encyclopedia of genes and genomes analyses

**Fig. 11:**
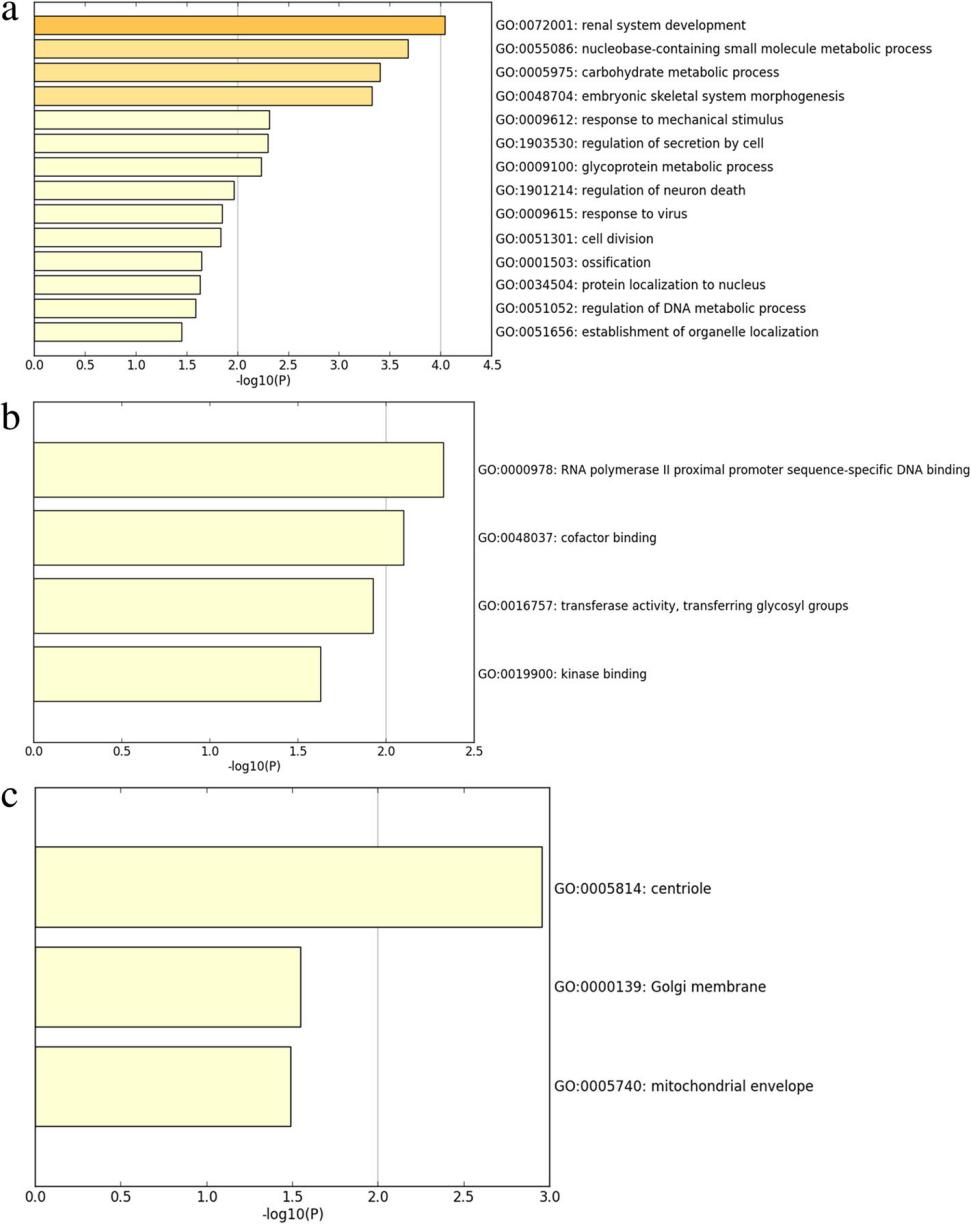
Distribution of gene ontology terms for the genes targeted by microRNA-144-3p in non-small cell lung cancer. **a** Biological process. **b** Molecular function. **c** Cellular component

**Fig. 12:**
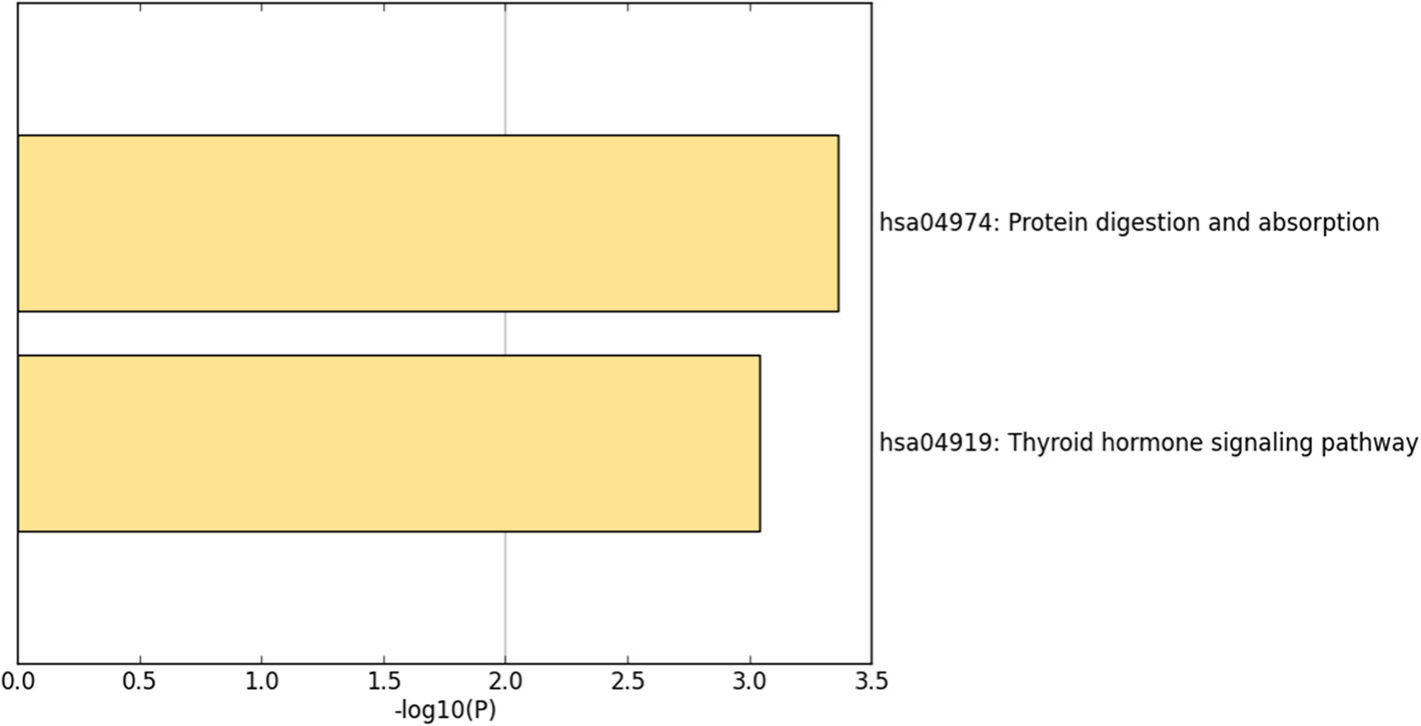
Distribution of Kyoto Encyclopedia of Genes and Genomes terms for the target genes of microRNA-144-3p in non-small cell lung cancer. Note: The protein digestion and absorption and the thyroid hormone signaling pathways were the top two pathways most strongly enriched by the target genes

**Fig. 13:**
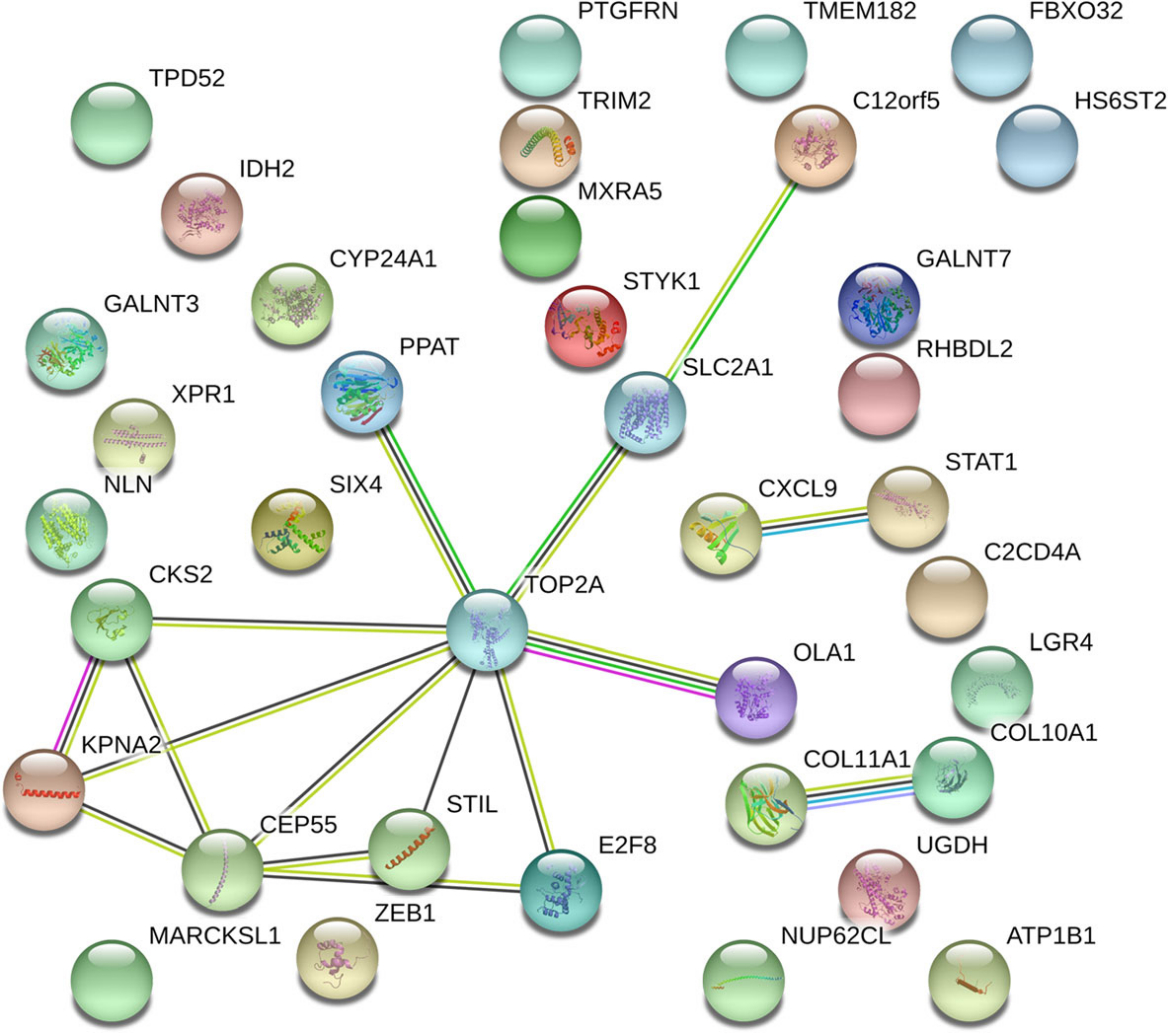
The protein–protein interaction networks of the promising target genes of microRNA-144-3p in non-small cell lung cancer. Notes: Edges represent protein–protein associations, and colored nodes represent query proteins and the first shell of interactors

For further interpretation of the function of the promising genes targeted by miR-144-3p in NSCLC, KEGG, and GO annotations were performed in Metascape. For the GO analysis, three categories were used: BP, CC, and MF. For the BP, renal system development (GO: 0072001) and the nucleobase-containing small molecule metabolic process (GO: 0055086) were the top two pathways (Fig. 11a). For the CC, the potential target differentially expressed genes (DEGs) were predominantly enriched in the centriole (GO: 0005814), Golgi membrane (GO: 0000139), and mitochondrial envelope (GO: 0005740) (Fig. 11c). For MF, the three significantly involved items were RNA polymerase II proximal promoter sequence-specific DNA binding (GO: 0000978); cofactor binding (GO: 0048037); and transferase activity, transferring glycosyl groups (GO: 0016757) (Fig. 11b). Regarding KEGG, the top two enriched pathways were the protein digestion and absorption (hsa04974) and the thyroid hormone signaling pathways (hsa04919) (Fig. 12). PPI revealed five genes—C12orf5, CEP55, E2F8, STIL, and TOP2A—as hub genes with the threshold value of 6 (Fig. 13).

### Expression of hub genes from the Cancer genome atlas and the genotype-tissue expression datasets

**Fig. 14:**
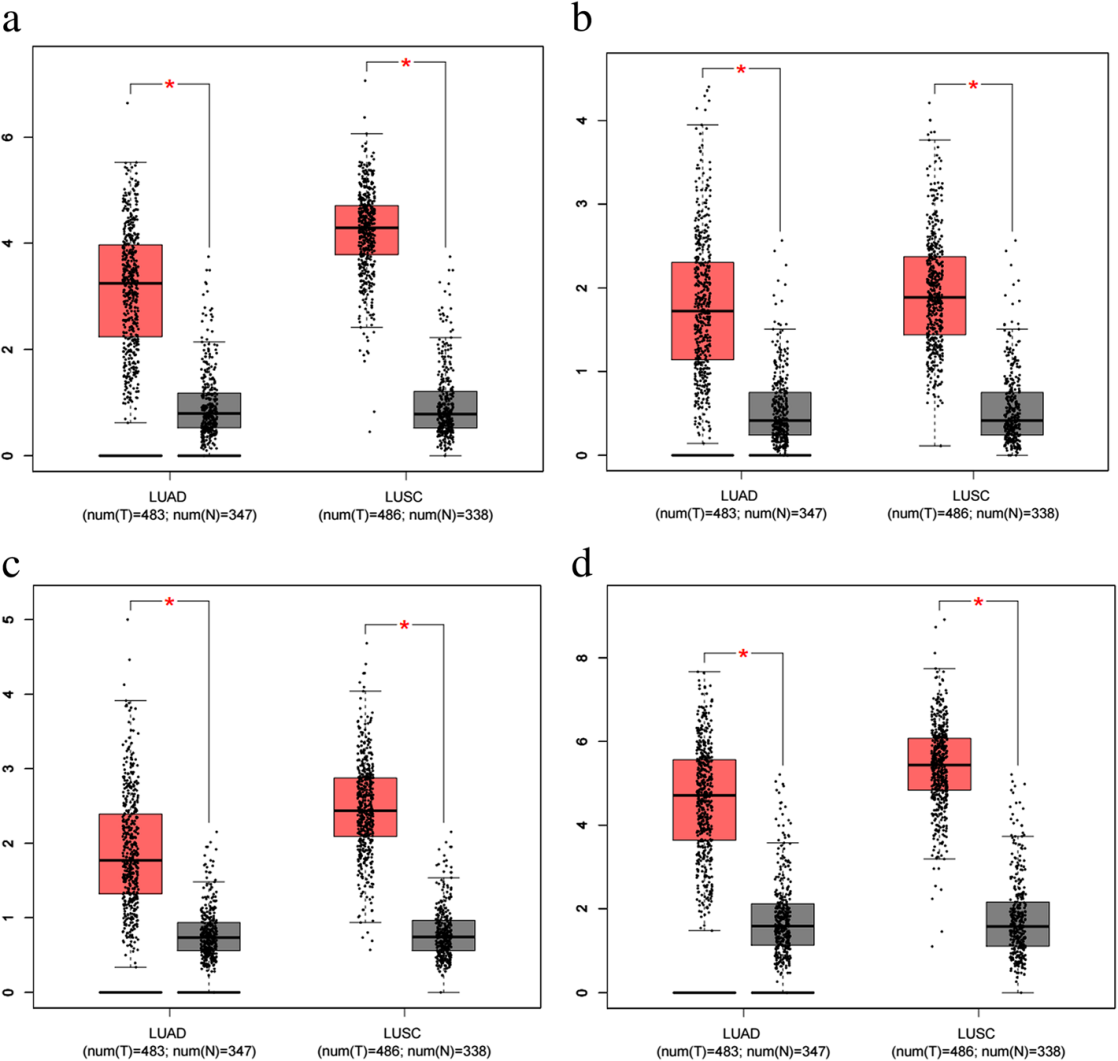
Expression of hub genes in non-small cell lung cancer and normal tissues, based on Gene Expression Profiling Interactive Analysis (GEPIA). Notes: Expression of the hub genes was detected in 969 NSCLC tissues (T) and 685 normal tissues (N) on the basis of GEPIA. Four of the genes—**a** CEP55, **b** E2F8, **c** STIL, and **d** TOP2A—were overexpressed in non-small cell lung cancer tissues when compared to the normal tissues

Of the five hub genes, not including C12orf5, that occupied the central region of the PPI network, four genes (CEP55, E2F8, STIL, and TOP2A) were significantly up-regulated in the NSCLC group compared to the control group (Fig. 14).

## Discussion

Although previous studies have documented the expression of miR-144-3p in NSCLC, correlations of the clinical features with NSCLC and miR-144-3p have been seldom reported. This study used a larger number of samples for a systematic investigation of the relationships. A highlight of this study was the use of the computational biology method to explore the latent mechanism of miR-144-3p in NSCLC.

A decreased expression of miR-144-3p was found in the NSCLC cases, as presented by the GEO, TCGA, and RT-qPCR. A comprehensive meta-analysis was the focus of the current study. Data were obtained from multiple sources: namely, microarrays, previous studies, RT-qPCR, and TCGA. The meta-analysis results revealed that the decline of miR-144-3p in NSCLC was consistent across studies [19, 22]. It was therefore concluded that there was a considerable decrease of miR-144-3p expression in NSCLC. Based on the data from TCGA, the miR-144-3p expression level in LUSC and LUAD was related to stage, and the predominant difference for T was found in LUAD only.

The results revealed that miR-144-3p could be involved in the occurrence and development of NSCLC, and low miR-144-3p could indicate the promotion of NSCLC. Regarding RT-qPCR, miR-144-3p expression was related to lymph node metastasis and vascular invasion. Moreover, the patients with lymph node metastasis and vascular invasion had a tendency to down-regulate miR-144-3p expression. Regarding LUAD, the amount of miR-144-3p differed greatly depending on the smoking status of the patient, the existence of lymph node metastasis, and the presence of vascular invasion. However, no statistical differences were found for LUSC. In sum, miR-144-3p might serve as a marker to monitor the progression of NSCLC.

No differences were observed in the survival times for the low-miR-144-3p group and the high-miR-144-3p group, according to TCGA data. In contrast, the PCR results revealed a trend that suggests that LUAD patients with lower miR-144-3p levels might have worse outcomes although not to a significant extent. A study by Wu et al. [23] revealed that miR-144-3p was one of the independent prognostic risk factors for LUAD patients, with those with low miR-144-3p having poor prognoses. Further investigations are required for corroboration.

At the present time, the specific molecular mechanism of NSCLC is not widely understood. Therefore, bioinformatics analyses were performed to discover the inherent mode of NSCLC activity at the molecular level. Based on miRWALK2.0 and TCGA data, the candidate targets of miR-144-3p were projected. In an attempt to explore the roles of these genes further, KEGG and GO annotation analyses were performed. According to GO enrichment, the candidate targets of miR-144-3p might have an important effect on the progression of NSCLC by modulating several cellular biology processes, such as renal system development. Moreover, these genes could also have an important effect, such as cofactor binding, on MF. The results of the KEGG analysis also showed the roles of the candidate targets of miR-144-3p in NSCLC. The top two enriched pathways were the protein digestion and absorption and the thyroid hormone signaling pathways. This suggests that the promising targets of miR-144-3p could be involved in the aforementioned pathways to influence the occurrence and progression of NSCLC.

The protein digestion and absorption pathway is a key pathway in several human cancers. A study by Shi et al. showed that this pathway might contribute to the pulmonary metastasis of osteosarcoma patients [24]. It might be involved in the up-regulation of differentially expressed genes in breast cancer [25], and it has already been associated with the down-regulated differentially expressed genes in pancreatic neuroendocrine tumors [26]. The protein digestion and absorption pathway is connected mainly to differentially expressed genes, and this affects the occurrence and development of enchondromas [27]. Additionally, B-cell malignancies are relevant to the protein digestion and absorption pathway [28]. However, few studies have been conducted on the protein digestion and absorption pathway in NSCLC. Therefore, more studies are required to further validate the specific molecular mechanism in NSCLC.

According to the PPI network data, five genes (TIGAR, CEP55, E2F8, STIL, and TOP2A) have been recognized as hub genes in NSCLC, thus proving their roles as ideal candidates for miR-144-3p targets. The current study found that four of the 5 hub genes—CEP55, E2F8, STIL, and TOP2A—were more greatly upregulated in the NSCLC cases than in the normal cases. In addition, the decreased miR-144-3p expression in NSCLC was validated in this study. Therefore, the overexpression of the four hub genes in NSCLC was indirect proof that these genes might be the targets of miR-144-3p. By targeting the TIGAR, miR-144-3p suppresses proliferation, mediates programmed cell death, and increases autophagy in LC cells [18]. Studies have found that CEP55 might have an important effect on the proliferation of LC [29, 30]. The role of the E2F8 gene has been studied in several cancers. Sun et al. [31] provided evidence that E2F8 was targeted by miR-144-3p in papillary thyroid cancer; however, a connection between E2F8 and miR-144-3P in NSCLC has not been reported so far.

The current study has limitations. Because a large-scale clinical sample was not used, more investigations with large clinical samples are required for further confirmation of the function of miR-144-3p in NSCLC prognoses. In addition, experiments were not conducted for the detection of the expression of hub genes. The expression of these genes needs to be verified by additional well-designed studies. Although bioinformatics analyses were performed, the specific molecular mechanisms were not identified.

In conclusion, the current study validated that miR-144-3p was lowly expressed in NSCLC. More importantly, miR-144-3p might function as a latent tumor biomarker in the prognosis prediction for NSCLC. The results of bioinformatics analyses may present a new method for investigating the pathogenesis of NSCLC.

## Abbreviations

BP: Biological process
CC: Cellular component
CI: Confidential interval
GEO: Gene Expression Omnibus
GO: Gene Ontology
KEGG: Kyoto Encyclopedia of Genes and Genomes
LC: Lung cancer
LCC: Lung large cell carcinoma
LUAD: Lung adenocarcinoma
LUSC: Lung squamous cell carcinoma
M: Mean
MF: Molecular function
NSCLC: Non-small cell lung cancer
PPI: Protein–protein interaction network
qRT-PCR: Quantitative real-time PCR
SD: Standard deviation
SMD: Standardized mean difference
TCGA: The Cancer Genome Atlas
